# Couple’s Satisfaction among Lebanese adults: validation of the Toronto Alexithymia Scale and Couple Satisfaction Index-4 scales, association with attachment styles and mediating role of alexithymia

**DOI:** 10.1186/s40359-022-00719-6

**Published:** 2022-01-19

**Authors:** Yara El Frenn, Marwan Akel, Souheil Hallit, Sahar Obeid

**Affiliations:** 1 Faculty of Medicine and Medical Sciences, Holy Spirit University of Kaslik (USEK), Jounieh, Lebanon; 2 School of Pharmacy, Lebanese International University, Beirut, Lebanon; 3 Research Department, Psychiatric Hospital of the Cross, Jal Eddib, Lebanon; 4 Social and Education Sciences Department, School of Arts and Sciences, Lebanese American University, Jbeil, Lebanon

**Keywords:** Attachment styles, Couple satisfaction, Alexithymia, Relationship satisfaction, Validation, Toronto Alexithymia Scale, Couple Satisfaction Index-4, Depression, Lebanon

## Abstract

**Background:**

Lebanon is passing through an economic crisis leading to a decreased monthly income within a couple and more couple’s dissatisfaction. Furthermore, many studies postulate that the different types of attachment styles affect the romantic relationship experienced between adults. The main objectives of our study were to (1) validate the Toronto Alexithymia Scale (TAS) and Couple Satisfaction Index-4 (CSI-4) scales, and (2) assess the association between attachment styles and couple satisfaction and evaluate the mediating role of alexithymia in these associations.

**Methods:**

This cross-sectional study involved 445 Lebanese participants (April–May 2021). A confirmatory factor analysis (CFA) was carried out using SPSS AMOS v.24 on the couple satisfaction index and Toronto alexithymia scales’ items. A linear regression was conducted, taking the couple satisfaction index as the dependent variable.

**Results:**

The CFA results of the CSI-4 scale indicated an excellent fit (χ^2^/df = 3.845/2 = 1.92, TLI = 0.992, RMSEA = 0.046 [95% CI 0.001–0.115] (pclose = 0.436) and CFI = 0.998). The CFA of the TAS indicated a good/acceptable fit (χ^2^/df = 422.31/132 = 3.2, TLI = 0.89, RMSEA = 0.07 [95% CI 0.063–0.078] (pclose < 0.001) and CFI = 0.91) (items 4 and 8 were removed due to low factor loading (< 0.4)). Being married and higher secure attachment style were significantly associated with more couple satisfaction, whereas older age, higher household crowding index, more alexithymia and mode depression were significantly associated with less couple satisfaction. In addition, alexithymia mediated the association between secure attachment style and couple satisfaction, between preoccupied attachment style and couple satisfaction and between dismissing attachment style and couple satisfaction.

**Conclusion:**

Couple’s satisfaction is positively associated with secure attachment style. In addition, alexithymia plays a mediating role between couple’s satisfaction and attachment styles. Upcoming studies should determine if other mental illnesses play a mediating role between attachment style and couple satisfaction.

## Background

Couple satisfaction is the subjective evaluation of one’s relationship; it is interpreted by the assessment of the positive feelings for one’s partner, the satisfaction with the relationship and its overall appraisal [1]. It is affected by many factors such as depression [2], relationship education [3], sexual communication [4], sexual satisfaction [5] and individual’s educational level [6]. Culture diversity is another factor that influences relationship satisfaction; collectivistic culture, similar to the Lebanese one, is characterized by fidelity, support and partnership that impact the couple’s relation. In contrast, in individualistic culture like the one present in Western countries, satisfaction is not correlated with meeting individual’s obligations but rather is attained when the couple’s goals are accomplished [7].

Moreover, depression, a major cause of disability worldwide [8], is associated with couple’s satisfaction [2]. As stated by the World Health Organization (WHO), more than 300 million people are affected by depression [9]. A study showed that 59.7% of the Lebanese population suffer from depression [10], and other mental health illnesses such as anxiety [11] and suicidal ideation [12]. Instability in the couple satisfaction was shown to be related to depressive symptoms severity [2].

Additionally, attachment styles affect couple satisfaction [13]. It presupposes that the primary relationship with the parents, starting from meeting one’s needs and developing a response mechanism, impacts the way of communication with the outer world [14]. In fact, attachment styles are known as an inborn human potential to correlate with one’s caregiver; hence, young children need to develop a relationship with at least one primary caregiver for a healthy social and emotional development [15]. From here, interpersonal bonds in adulthood are vigorously affected and determined by the attachment relationship background [16]. The most significant part of the attachment theory elucidates that people unconsciously generalize what has been already acquired in the early years; the bonding established between two persons in the early stage of development, affects the quality of bonding later in adulthood [17, 18]. Bartholomew and Horowitz classified attachment styles into four different categories: secure, preoccupied, dismissive and fearful [19]. Securely attached individuals are usually known to be self-confident, trusting, hopeful, and responsive to intimate relationship. Fearful persons may find it difficult to connect to others, or are anxious thinking they will face failure or be rejected by their partner. For people with the preoccupied type, they are often anxious and uncertain, and lack self-esteem as they worry that they might be unwanted by others. For the dismissive type, the person is so wary of closeness and try to avoid emotional connection with others, thus, may not look for a relationship [19]. A constructive association between secure attachment and couple satisfaction was demonstrated by most researchers, and a negative correspondence between insecure attachment and couple satisfaction [20–23]. Some people find the idea of connecting and bonding with others distressing and uncomfortable; this falls under the category of social phobia and anxiety illness [24]. Previous studies in Lebanon tackled the relationship between attachment styles and alcohol use disorder [25, 26], mental health illnesses such as alexithymia, anxiety, depression [27] and fear of intimacy [28], but none of them tackled their relationship with couple’s satisfaction.

According to the type of attachment style, one can discover the degree of emotional regulation; thus, people with insecure attachment style have greater levels of alexithymia [29]. Each of the partners should acknowledge and value the feelings of each other in order to maintain a healthy couple relationship. Hence, individuals with alexithymia are negatively associated with couple’s satisfaction [30]. Alexithymia is characterized by the inability to identify and describe emotions experienced by one’s self [31, 32]. People with alexithymia find it hard to control their emotions which are linked with low levels of social ability, emotion expression and intelligence [33]. The percentage of alexithymia was recorded high in Lebanon as 20.8% of the population suffer from this illness [34]. Alexithymia has a moderator role between attachment styles and couple satisfaction: this disorder reduces indirectly the relationship satisfaction [35] and is linked to the fearful and preoccupied styles rather than the secure attachment style [36, 37]. A Lebanese study estimated the factors that are linked with alexithymia among the population; and came to a realization that tension, emotional and mental fatigue, alcohol addiction and anxiety are linked with more alexithymia, but being married is linked with less alexithymia [34]. Another study conducted in Lebanon showed that insecure attachment styles are linked positively to alexithymia, in opposite to the secure one that is negatively linked to alexithymia [27].

Lebanon is passing through an economic crisis [38] leading to a decreased monthly income within a couple and more couple’s dissatisfaction [39]. Furthermore, many studies postulate that the different types of attachment styles affect the romantic relationship experienced between adults [40, 41]. Also, there are no studies in Lebanon about that subject and about the mediating role of alexithymia in these associations. The main objectives of our study were to (1) validate the Toronto Alexithymia Scale (TAS) and Couple Satisfaction Index-4 (CSI-4) scales, and (2) assess the association between attachment styles and couple satisfaction and evaluate the mediating role of alexithymia in these associations. We hypothesize that secure attachment style would be linked with higher couple satisfaction, in opposite to insecure attachment styles; and that alexithymia would increase couple’s dissatisfaction in individuals with insecure attachment styles.

## Methods

### Study design and participants

This cross-sectional study involved 445 Lebanese participants between April and May 2021. The snowball technique was followed during the data collection; a Google form was designed and distributed via social media to persons from all Lebanese districts (Beirut, Bekaa, Mount Lebanon, South Lebanon and North Lebanon). These persons were asked to forward that link to other friends they know. All participants were informed about the objective of this study and the anonymity of participation. Participants had the freedom to accept or decline the invitation, with no monetary compensation received for participation. All methods were carried out in accordance with relevant guidelines and regulations.

### Minimal sample size calculation

The G-power system was used to calculate the minimal sample size required based on an alpha error of 5%, a power of 80% and 12 factors to be entered in the multivariable analysis. The minimal sample size required was 395 participants.

### Ethical approval

This study protocol was approved by the Psychiatric Hospital of the Cross Ethics and Research Committee (HPC-020-2021). Submitting the form online was considered equivalent to obtaining a written consent.

### Questionnaire

This survey was created in the native language of Lebanon (Arabic). Twenty minutes were required to complete the form. It was divided to several sections:

#### Sociodemographic characteristics

This section included questions about individuals’ age, gender, educational level, marital status and Household Crowding Index (HCI). The HCI was obtained by dividing the number of persons living in the house by the number of rooms in the house [42].

#### Couple Satisfaction Index-4 (CSI-4)

It is composed of 4-items to measure the relationship satisfaction in couples [43]. Each item is graded from 0 (not at all) to 5 (absolutely). The higher the score, the higher the satisfaction of the couple. The Cronbach’s alpha in this study was 0.872.

#### Toronto alexithymia scale (TAS 20)

Alexithymia was evaluated by using the 20-item Toronto Alexithymia Scale [44]. It has an adequate validity and credibility [45, 46]. Each item is calculated by using the 5-point Likert scale (from 1 = strongly disagree to 5 = strongly agree). Professor Graeme Taylor gave us the permission to use this scale and provided us with its Arabic version. The Cronbach’s alpha in this study was 0.913.

### The relationship questionnaire

Each item describes one of the four types of the adult attachment styles: secure (style A), preoccupied (style B), fearful (style C), and dismissing (style D) [47]. Each item is graded on a 7-point scale (1 = strongly disagree to 7 = strongly agree). The Cronbach’s alpha in this study was 0.76.

#### Lebanese Depression Scale (LDS-19)

This is a 19-item scale, validated in Lebanon and used to assess the symptoms and signs of depression among the Lebanese population [48]. The higher the score, the higher the depression. The Cronbach’s alpha in this study was 0.921.

### Translation procedure

The translation from English to Arabic was carried out by a single bilingual translator for the couple satisfaction index scale and relationship questionnaire. A backward translation was then performed by another translator, fluent in Arabic and unfamiliar with the concepts of the scales. Discrepancies were resolved by consensus between translators and researchers.

### Statistical analysis

A confirmatory factor analysis (CFA) was carried out using SPSS AMOS v.24 on the couple satisfaction index and Toronto alexithymia scales’ items. The root mean square error of approximation (RMSEA) statistic, the Tucker Lewis Index (TLI) and the comparative fit index (CFI) were used to evaluate the goodness-of-fit of the model as these are the most commonly used indices [49]. Values of RMSEA of 0.06 or less indicate a good-fitting model and a value larger than 0.10 is indicative of a poor model [49], while TLI and CFI values greater than 0.90 indicate excellent model fit [49], whereas TLI values ≥ 0.85 [49] and CFI values > 0.80 [50] indicate good model fit.

The SPSS software v.25 was used for all statistical analysis. The normality of distribution of the couple satisfaction index was confirmed via a calculation of the skewness and kurtosis; values for asymmetry and kurtosis between − 1 and + 1 are considered acceptable in order to prove normal univariate distribution [51]. These conditions consolidate the assumptions of normality in samples larger than 300 [52]. The Student t test was used to test for an association between the score and dichotomous variables. Finally, the Pearson correlation test was used to correlate two continuous variables. A linear regression was conducted, taking the couple satisfaction index as a dependent variable.

The PROCESS SPSS Macro version 3.4, model four [54] was used to calculate three pathways. Pathway A determined the regression coefficient for the effect of each attachment style on alexithymia (mediator); Pathway B examined the association between alexithymia and couple’s satisfaction, and Pathway C’ estimated the total and direct effect of each attachment style and couple’s satisfaction. A mediation was deemed significant if the bootstrapped 95% confidence intervals of the indirect pathway AB did not pass by zero. Variables that showed a p < 0.25 in the bivariate analysis were taken as independent ones in the linear and mediation models [53].^1^ p < 0.05 was deemed statistically significant.

## Results

The sample consisted of 445 participants, with a mean age of 28.38 ± 13.26 years and 67.2% females. Other characteristics and description of the scores can be found in Table 1.

**Table 1:** Sociodemographic characteristics of the participants (N = 445)

Variable	N (%)
*Gender*	
Male	146 (32.8%)
Female	299 (67.2%)
*Marital status*	
Single (but in a serious relationship)	321 (72.2%)
Married	124 (27.8%)
*Education level*	
Secondary or less	321 (72.2%)
University	124 (27.8%)

	Mean ± SD
Age (in years)	28.38 ± 13.26
Number of children	1.13 ± 1.98
Household crowding index	1.33 ± 0.94
Alexithymia	57.11 ± 9.22
Depression	15.77 ± 12.50
Couple satisfaction index	9.69 ± 5.47

### Confirmatory factor analysis of the couple satisfaction index

The following results were obtained: the Maximum Likelihood Chi-Square = 3.845 and Degrees of Freedom = 2, which gave a χ^2^/df = 1.92. The TLI value was 0.992. The RMSEA value was 0.046 [95% CI 0.001–0.115] (pclose = 0.436) and CFI value was 0.998 respectively, indicating an excellent fit of the model. Table 2 presents the coefficients with standard errors and p-values of the direct effects of variables on each other.

**Table 2:** Item descriptive statistics, standardized factor loadings, and explained variance of the couple satisfaction index

Variable	Standardized factor loadings	Standard error	*p*
CSI 1	1		
CSI 2	0.84	0.13	< 0.001
CSI 3	0.93	0.14	< 0.001
CSI 4	0.91	0.14	< 0.001

### Confirmatory factor analysis of the Toronto Alexithymia Scale

Items 4 and 8 were removed due to low factor loading (< 0.4). The following results were obtained for the remaining items: the Maximum Likelihood Chi-Square = 422.31 and Degrees of Freedom = 132, which gave a χ^2^/df = 3.2. The TLI value was 0.89. The RMSEA value was 0.07 [95% CI 0.063–0.078] (pclose < 0.001) and CFI value was 0.91 respectively, indicating a good/acceptable fit of the model. Table 3 presents the coefficients with standard errors and p-values of the direct effects of variables on each other.

**Table 3:** Item descriptive statistics, standardized factor loadings, and explained variance of the couple satisfaction index

Variable	Standardized factor loadings	Standard error	*p*
*Factor 1*			
TAS 2	1		
TAS 11	0.718	0.076	< 0.001
TAS 12	0.596	0.072	< 0.001
TAS 17	0.509	0.077	< 0.001
*Factor 2*			
TAS 1	1		
TAS 3	0.573	0.066	< 0.001
TAS 6	0.680	0.072	< 0.001
TAS 7	0.762	0.068	< 0.001
TAS 9	0.780	0.069	< 0.001
TAS 13	0.698	0.068	< 0.001
TAS 14	0.651	0.070	< 0.001
*Factor 3*			
TAS 5	1		
TAS 10	0.724	0.097	< 0.001
TAS 15	0.419	0.082	< 0.001
TAS 16	0.480	0.088	< 0.001
TAS 18	0.693	0.096	< 0.001
TAS 19	0.743	0.097	< 0.001
TAS 20	0.425	0.087	< 0.001

### Bivariate analysis

Higher alexithymia, depression, and household crowding index were significantly associated with lower couple satisfaction index, whereas higher secure and fearful attachment styles were significantly associated with more couple satisfaction index (Table 4). Furthermore, married participants and those with a university level of education had significantly higher couple satisfaction index than single and those with a secondary level of education or less respectively (Table 5).

**Table 4:** Correlation between the couple satisfaction index and other continuous variables

Variable	Correlation coefficient	*p*
Alexithymia	− 0.197	**< 0.001**
Depression	− 0.195	**< 0.001**
Secure attachment style	0.196	**< 0.001**
Preoccupied attachment style	− 0.033	0.487
Fearful attachment style	0.115	**0.015**
Dismissing attachment style	− 0.025	0.592
Age	− 0.092	0.054
Number of children	− 0.011	0.816
Household crowding index	− 0.193	**< 0.001**

Numbers in bold indicate significant p-values

**Table 5:** Correlation between the couple satisfaction index and other categorical variables

Variable	Mean ± SD	*p*	Effect size
*Gender*		0.990	0
Male	9.69 ± 5.36		
Female	9.69 ± 5.54		
*Marital status*		**< 0.001**	0.498
Single (but in a serious relationship)	8.96 ± 5.41		
Married	11.60 ± 5.19		
*Education level*		**0.034**	0.259
Secondary or less	9.42 ± 5.48		
University	10.82 ± 5.32		

The Student t test was used to compare two means

### Multivariable analysis

The results of a linear regression using the ENTER model and taking the couple’s satisfaction index as the dependent variable, showed that being married (Beta = 0.29) and higher secure attachment style (Beta = 0.16) were significantly associated with more couple satisfaction, whereas older age (Beta = − 0.28), higher household crowding index (Beta = − 0.22), more alexithymia (Beta = − 0.16) and mode depression (Beta = − 0.12) were significantly associated with less couple’s satisfaction (Table 6).

**Table 6:** Multivariable analysis: linear regression taking the couple satisfaction index as the dependent variable

Variable	Unstandardized beta	Standardized beta	*p*	95% confidence interval
Marital status (married vs single*)	3.59	0.29	**< 0.001**	2.38 to 4.81
Education level (university vs secondary or less*)	0.23	0.02	0.699	− 0.95 to 1.42
Age	− 0.12	− 0.28	**< 0.001**	− 0.16 to − 0.07
Household crowding index	− 1.29	− 0.22	**< 0.001**	− 1.79 to − 0.79
Alexithymia	− 0.09	− 0.16	**0.001**	− 0.15 to − 0.04
Depression	− 0.05	− 0.12	**0.009**	− 0.09 to − 0.01
Secure attachment style	0.47	0.16	**0.002**	0.17 to 0.78
Preoccupied attachment style	− 0.27	− 0.09	0.137	− 0.62 to 0.09
Fearful attachment style	0.27	0.08	0.139	− 0.09 to 0.62
Dismissing attachment style	− 0.21	− 0.06	0.221	− 0.56 to 0.13

*Reference group

### Mediation analysis

The detailed results of the mediation analysis between attachment styles, alexithymia and couple satisfaction are summarized in Table 7. The results showed that alexithymia mediated the association between secure attachment style and couple satisfaction by 19.19%, between preoccupied attachment style and couple satisfaction by 27.80% and between dismissing attachment style and couple satisfaction by 17.32%.

**Table 7:** Mediation analysis

	Effect of attachment style on alexithymia					Effect of attachment style and alexithymia on couple satisfaction index					Effect of attachment style on couple satisfaction index					Mediating effect of alexithymia
	Beta	95% BCa CI		t	p	Beta	95% BCa CI		t	p	Beta	95% BCa CI		t	p	
Model 1: Attachment style, alexithymia and couple satisfaction index																
Secure	− 0.94	− 1.46	− 0.42	− 3.53	**< 0.001**	0.56	0.25	0.86	3.57	**< 0.001**	0.66	0.36	0.97	4.24	**< 0.001**	19.19%
Alexithymia						− 0.11	− 0.17	− 0.06	− 4.11	**< 0.001**						
Preoccupied	0.99	0.45	1.54	3.57	**< 0.001**	− 0.37	− 0.69	− 0.04	− 2.22	**0.026**	− 0.47	− 0.79	− 0.15	− 2.85	**0.004**	27.80%
Alexithymia						− 0.10	− 0.16	− 0.05	− 3.69	**< 0.001**						
Fearful	0.86	0.34	1.38	3.23	**0.001**	− 0.07	− 0.38	0.24	− 0.44	0.661	− 0.17	− 0.48	0.14	− 1.06	0.290	–
Alexithymia						− 0.11	− 0.17	− 0.06	− 4.11	**< 0.001**						
Dismissing	0.41	− 0.09	0.91	1.60	0.111	− 0.26	− 0.55	0.03	− 1.75	0.08	− 0.30	− 0.60	− 0.01	− 2.03	**0.043**	17.32%
Alexithymia						− 0.11	− 0.17	− 0.06	− 3.97	**< 0.001**						

Numbers in bold indicate significant p-values

## Discussion

Our study results showed that being married and having higher secure attachment style were positively associated with more couple satisfaction. However, older age, higher household crowding index, having more alexithymia and depressive symptoms were associated with less couple satisfaction. Moreover, alexithymia mediated the associations between secure, preoccupied and dismissing attachment styles and couple satisfaction.

### Attachment styles and couple satisfaction

Higher secure attachment style was significantly associated with more couple satisfaction, in line with previous studies [55, 56]. In fact, individuals with secure attachment style are characterized by higher self-esteem, trust in others [57–59] and are comfortable expressing their affection for their partner [55]. This positive attitude a person holds toward himself/herself and others increases couple satisfaction, leading to a healthy communication with one another [55]. A healthy couple life is generated by a purposeful bonding between the two of them as they face stressful events with compassion and support by performing acceptance and consensus [60].

### Alexithymia and couple satisfaction

Similar to a previous study [30], our results showed that alexithymia was negatively associated with couple’s satisfaction. This result proposes that discomfort in emotional closeness is usually reflected by relationship dissatisfaction, which is associated with more alexithymia [61]. This difficulty in recognition of emotion outcomes in the lack of clear uttering of feelings and compliance between the couple as it initiates anger and anxiousness which maximizes couple conflicts [60].

### Depression and couple satisfaction

Furthermore, depression was negatively associated with couple’s satisfaction in our study, in line with previous findings [2, 62]. Depressed individuals are more prone to negative thoughts, thus, in relationships, they consider themselves as rejected and despicable in the eyes of their partner who they think are unsupportive and discouraging [63].

### Mediating role of alexithymia

This study showed that alexithymia played a mediating role between couple satisfaction and different types of the attachment style. Relationship quality is deeply associated with its emotional aspects as it is differently evaluated and defined by people with secure and insecure attachment styles. Secure people express intimacy, love and friendship, while insecure ones have a contradiction and a pessimistic view of a relationship as they consider it as emotionally distancing and anxious [60]. Therefore, individuals with insecure attachment styles lack the capacity to live their own real sentiments, feel that happiness is unreachable to them and having an optimistic view of life seems impossible [60]; the opposite is true about people with a secure attachment style [60]. The capacity of setting and keeping up a romantic relationship needs the identification of emotions and their expressions, as well as the understanding of others’ feelings [64–66]. Therefore, alexithymia has a positive relationship with difficulty in communication and bonding with others [67].

### Sociodemographic characteristics

In line with a previous study [68], being married is associated with more couple satisfaction. This result shows that in a relationship, gladdening each other’s need for love, glorifying togetherness, familiarity and daily interaction, help boost couple’s satisfaction [69].

In line with a previous study [70], having a higher crowding index is associated with lower couple’s satisfaction. Economic stress and low levels of economic well-being may exacerbate financial conflicts and lower gratifications as couples struggle to make ends meet [70]. However, some poor couples adjust well to their restricted assets, while some richer couples might in any case have periodic hardships taking care of their financial obligations [70].

Our study showed that being older was negatively associated with couple’s satisfaction. No studies have mentioned this relationship between the two variables. We hypothesize that individuals with older age face more stressful events (such as retirement, decreased income, etc.), which can affect their living condition and consequently lead to less couple’s satisfaction. However, further studies are needed to show the exact cause of this association.

### Limitations and suggestions for future research

Some limitations are present in this study. The cross-sectional type used cannot demonstrate causation. Sampling bias exists since the data was collected through a snowball technique via social media. The questionnaire was self-administered, thus information bias is present because the variables were not assessed by a healthcare professional. Our study did not include the length of the relationship length, which has been proved to be associated with couple’s satisfaction [71]. The results of this study should be interpreted with caution since the four paragraphs of the attachment styles questionnaire contain several sentences and participants do not always how to respond, which affects the validity of the questionnaire [71]. Also, other variables associated with couple’s satisfaction that were not assessed in this study, may lead to confusion bias. However, we think that our results are solid enough since they rely on validated scales. Upcoming studies should determine if other mental illnesses play a mediating role between attachment style and couple satisfaction.

## Conclusion

Couple’s satisfaction was positively associated with secure attachment style. In addition, alexithymia played a mediating role between couple’s satisfaction and attachment styles, which highlights the need to stage interventions to reduce alexithymia as well as engage social support. However, the mediating role of alexithymia varied according to the different types of attachment styles. Improving people’s coping mechanisms and resilience when facing their problems (such as the economic crisis in our current situation in Lebanon, etc.) in general and in alexithymic individuals and those with insecure attachment styles in particular, might help them decrease distress, which in turn can improve their couple’s satisfaction [72]. Furthermore, alexithymic individuals can benefit from psychological therapeutic methods to improve their social connectedness (the awareness of an individual that he/she is engaged in a social relationship), thus, leading to a positive personality and increasing life satisfaction [73].

## Data Availability

The authors do not have the right to share any data information as per their institutions policies.

## Abbreviations

WHO: World Health Organization
FOI: Fear of intimacy
TAS-20: Toronto Alexithymia Scale (TAS 20)
CSI-4: Couple Satisfaction Index-4
LDS-19: Lebanese Depression Scale

## Footnotes

1 In the paper of Bursac et al., 2008 (link: https://scfbm.biomedcentral.com/articles/10.1186/1751-0473-3-17), authors state that variables that should be entered in a logistic regression model are the ones that show a p < 0.25. We contacted Pr. Hosmer and Pr. Lemeshow who said that these assumptions would likely hold for linear regression, but to date there is no published research examining this criteria for variable selection.
